# Processing and Bread-Making Quality Profile of Spanish Spelt Wheat

**DOI:** 10.3390/foods12162996

**Published:** 2023-08-09

**Authors:** Ana Belén Huertas-García, Carlos Guzmán, Maria Itria Ibba, Marianna Rakszegi, Josefina C. Sillero, Juan B. Alvarez

**Affiliations:** 1 Departamento de Genética, Escuela Técnica Superior de Ingeniería Agronómica y de Montes, Edificio Gregor Mendel, Campus de Rabanales, Universidad de Córdoba, CeiA3, ES-14071 Córdoba, Spain; b42hugaa@uco.es (A.B.H.-G.); jb.alvarez@uco.es (J.B.A.); 2 Global Wheat Program, International Maize and Wheat Improvement Center (CIMMYT), Apdo Postal 6-641, Mexico DF, Mexico; m.ibba@cgiar.org; 3 Agricultural Institute, Centre for Agricultural Research, Brunszvik u. 2, 2462 Martonvásár, Hungary; rakszegi.mariann@atk.hu; 4 IFAPA Alameda del Obispo, Avenida Menéndez Pidal s/n, 14004 Cordoba, Spain; josefinac.sillero@juntadeandalucia.es

**Keywords:** wheat quality, genetic resources, ancient wheat, bread-making

## Abstract

Spelt wheat (*Triticum aestivum* L. ssp. *spelta* Thell.) is an ancient wheat that has been widely cultivated for hundreds of years. Recently, this species has been neglected in most of Europe; however, the desire for more natural and traditional foods has driven a revival of the crop. In the current study, eighty-eight traditional spelt genotypes from Spain, together with nine common wheat cultivars and one modern spelt (cv. Anna Maria) were grown during a period of two years in Andalucia (southern Spain). In each, several traits were measured in to evaluate their milling, processing, and end-use quality (bread-making). The comparison between species suggested that, in general, spelt and common wheat showed differences for most of the measured traits; on average, spelt genotypes had softer grains, higher protein content (14.3 vs. 11.9%) and gluten extensibility (alveograph P/L 0.5 vs. 1.8), and lower gluten strength (alveograph W 187 vs. 438 × 10^−4^ J). In the baking test, both species showed similar values. Nevertheless, the analysis of this set of spelt genotypes showed a wide range for all measured traits, with higher values than common wheat in some spelt genotypes for some traits. This opens up the possibility of using these materials in future breeding programs, to develop either new spelt or common wheat cultivars.

## 1. Introduction

Since the 1960s, the importance of plant genetic resources has gradually increased, as shown by the development of the “International Treaty on Plant Genetic Resources for Food and Agriculture” [1]. The lack of genetic diversity in crops, globalization, and climate change have shown how easy is for any pathogen or plague to quickly spread around the world [2]. This could be a threat for food security, as modern agriculture is increasingly focused on fewer crops and fewer varieties within each crop [3]. At the same time, a greater awareness of the need to use more sustainable and environmentally friendly agronomic techniques, together with the problems associated with global change, have boosted the search for alternative gene sources, which is one of the strategies used to develop more resilient cultivars under the conditions of global warming. In this context, ancient wheats and wild-wheat relatives, which have adapted to be grown in marginal zones under extreme conditions [4], are considered to host interesting and unexploited genetic variability that could be used in modern wheat-breeding programs to develop more resilient cultivars.

Among these ancient wheats, spelt (*Triticum aestivum* L. ssp. *spelta* Thell., 2n = 6× = 42, A^u^A^u^BBDD), originally obtained from the natural hybridization between emmer wheat (*T. turgidum* spp. *dicoccum* Schrank em. Thell., = 4× = 28, A^u^A^u^BB) and *Aegilops tauschii* ssp. *strangulata* Coss. (2n = 2× = 14, DD) in the Fertile Crescent (Near East), is, today, the most cultivated species, and several spelt cultivars have been bred in order to improve their productivity [5,6]. For this reason, some ancestral traits like the hulled grain or the semi-branching rachis, have been modified through crosses with common wheat [7,8,9,10]. Consequently, two different types of spelt are now present in the farmers’ fields: the traditional or pure spelt, and the modern spelt derived from hybridization with common wheat [5]. The variability of these two types of spelt is notably different, with the traditional spelt holding a greater genetic variability than modern spelt. Nevertheless, more studies comparing the variability of modern and traditional spelt are needed.

On the other hand, several studies have suggested the exceptionality of the Iberian spelt (pol. *ibericum* Flaskb.) compared to the rest of the European spelt (Bavarian group, pol. *bavaricum* Vav.), including the old studies of N.I. Vavilov [11,12,13,14]. Therefore, while European spelt could derive from a secondary hybridization event between emmer wheat and hexaploid wheat, the Iberian spelt would have originated from the first hybridization event between emmer wheat and *Ae. tauschii* ssp. *strangulata* in Asia [11,12,13,14]. Furthermore, the spelt crop in Spain has been scarce until recent times and mainly based in traditional materials. The appearance of modern spelt in Spain is recent, and only two cultivars have been developed since 2018: cvs. Anna Maria and Viso. However, the traditional Spanish spelt stored in germplasm banks is abundant [14]. The current trend with this crop opens up the opportunity to add value to these old materials for their use in the current agricultural context, both as pure spelt and as a source of novel genetic diversity to develop modern spelt [14,15] or common wheat cultivars.

Before this new trend of growing old crops, spelt had already been used in breeding programs as a source of resistance genes for some wheat diseases [16,17,18,19,20,21,22,23,24,25,26]. Now, in the context of the renewed interest in artisan and “more natural” food, spelt is used as raw material for the making of food products (bread, biscuits, pasta, pancakes, etc.) which are present in many bakeries but also in large retailers. For this reason, studies on the processing quality of this crop have increased in importance [27,28,29,30,31,32,33,34].

Wheat processing quality is complex and varies depending on the wheat processor (millers or bakers) and on the final products. Wheat millers for example, value grain size, test weight, and texture, which are associated with the flour yield, and grain protein content, which is a partial indicator of wheat functionality [35,36]. Bakers, on the other hand, value the quantity and quality of the protein in flour, and the rheological properties of the dough made with it. For these reasons, the evaluation of new wheat materials must consider the quality requirements of all wheat processors including both millers and bakers.

Most of the studies conducted on the processing quality of ancient wheat only included a limited number of accessions [37] which reduced their ability to gain a clear understanding of the potential in terms of wheat processing, of such species. In general, the quality characteristics of these ancient wheats have been compared with modern wheat (common wheat–*T. aestivum* L. ssp. *aestivum*). Although this could be right, these data should be evaluated with caution. Many of these ancient wheats have been revived as a modern wheat substitute, and consequently, to establish the quality characteristics of modern wheat as the reference could be inadequate and lead to underestimating all ancient wheats. Obviously, spelt is not common wheat. The development of new cultivars of these ancient wheats should be complementary to modern wheat in the context of the new agri-food industry.

The main objective of this study was the evaluation of a wide collection of Spanish traditional spelt accessions for some grain and technological quality traits, together with their comparison with one modern spelt (cv. Anna Maria) and several common wheat cultivars widely cultivated in Andalucia (southern Spain).

## 2. Materials and Methods

### 2.1. Plant Material and Field Trials

Eighty-eight accessions of Spanish traditional spelt, together with ten modern wheat cultivars (nine common wheats and one modern spelt) were used (Table S1). These materials were planted in a randomized complete block design with two replicates during 2019–2020 and 2020–2021 crop seasons in Cordoba (Andalusia, Spain). Due to the high number of materials, the plot size was small (0.13 m^2^) and, consequently, the grain yield was limited for some accessions that could be only evaluated from small-scale tests.

The 88 traditional spelt accessions were selected according to their grain protein composition and origin from two wide collections originally provided by the National Small Grains Collection (USDA, Washington, DC, USA) and Centro de Recursos Fitogeneticos (INIA, Madrid, Spain) [38,39]. Of the 10 modern cultivars used as control, 9 were commercial Spanish common wheat cultivars commonly grown in the south of Spain (cvs. Antequera, Arthur Nick, Conil, Galera, Montemayor, Rota, Santaella, Setenil, and Tejada), and fall into different categories within the Spanish quality groups, depending on their performance in each environment. Cvs. Antequera, Conil, Galera, Rota, and Tejada often fall within the Spanish quality group 1 (strong gluten wheat for mechanized bread-making), while cvs. Arthur Nick, Montemayor, Santaella, and Setenil produce grains that are usually classified as quality groups 2–3 (strong–medium gluten wheat for semi-mechanized bread-making).The modern spelt (cv. Anna Maria) is a modern spelt cultivar obtained from hybridization between pure spelt and common wheat.

### 2.2. Grain and Flour Quality Traits

Thousand kernel weight (TKW, g) and test weight (TW, kg/hL) were obtained using the SeedCount SC5000 digital imaging system (Next Instruments, Australia). The grain (GPC, %) and flour (FPC, %) protein content were determined by near infrared spectroscopy (NIR Systems 6500, Foss, Hillerød, Denmark) based on AACC official methods 39-10.01 and 39-11.01, respectively, which were calibrated based on method 46-11.02 [40]. Grain hardness was measured on samples of 100 kernels with the single-kernel characterization system (SKCS) (Perten Instruments, Springfield, IL, USA) [40]. The polyphenol oxidase (PPO) activity was measured by absorbance at 475 nm according to Anderson and Morris [41], and expressed in Ug^−1^min^−1^.

For the milling, the two field replicates of each genotype were mixed in order to obtain enough flour. The grain samples were processed applying AACC method 26-95 [40]. All samples were milled into flour using a Brabender Quadrumat Senior mill (CW Brabender, Duisburg, Germany) and flour yield (%) was calculated.

Measurement of sodium dodecyl sulfate (SDS) sedimentation volume (ml) was carried out according to Dick and Quick methodology [42] with the modifications introduced by Peña et al. [43].

### 2.3. Alveographic and Baking Traits

The dough tenacity (P), extensibility (L), tenacity/extensibility ratio (P/L), tenacity/swelling index ratio (P/G), elasticity index (Ie), and strength (W) were determined by AACC method 54-30.02 using a Chopin alveograph [40]. Due to the limited flour available, dough rheological properties were measured only on 7 common wheat cultivars and 80 spelt accessions.

The bread-making process was conducted on 4 common wheat cultivars and 50 spelt accessions (only of those genotypes for which there was enough flour available to perform the test), using the direct dough method (AACC method 10-10.03), and loaf volume (cc) was determined by rapeseed displacement using a volume meter [40].

### 2.4. Statistical Methods

The comparison between both species sets was carried out for each trait analyzed by Student’s *t*-test. A Pearson correlation analysis was carried out among the grain, flour, and rheological traits within the Spanish spelt set.

For the spelt set, data were analyzed by an analysis of variance (ANOVA) using genotype, year, and genotype × year as variation sources. Because cv. Anna Maria represented the current trend in spelt, the cv. Anna Maria data for each measured traits were used as reference to evaluate and compare the values of each Spanish traditional spelt genotype.

All statistical analyses were performed using Statistix software (version 9).

## 3. Results

### 3.1. Comparison among Species

The data obtained for all materials evaluated (Tables S2 and S3) were grouped according to the species (common wheat vs. spelt) in order to compare the two groups. The mean values of each set were analyzed by Student’s *t*-test (Table 1).

For grain or flour components, the differences between both species were, in general, small, but significant. The thousand kernel weight (TKW) of spelt was slightly higher than common wheat; however, spelt grains showed lower test weight (TW) values, probably due to the morphology of their grains that have, on average, an elongated shape. This had no influence on the flour yield, although the grain hardness, clearly lower in spelt, could also have played a role on the flour yield.

The protein content was higher in spelt, both in grain and in flour. But this scarcely influenced the gluten strength measured by the SDS-sedimentation test, because the Student’s *t*-test analysis indicated that the differences between both species were not significant (Table 1). On the contrary, there were highly significant differences for polyphenol oxidase (PPO) activity, for which the spelt group exhibited the double mean activity of the common wheat group.

The alveographic parameters showed that while common wheat presented dough with high tenacity (P) and low/moderate extensibility (L), the spelt genotypes showed, in general, low to moderate tenacity (P) and high extensibility (L). In any case, the dough strength (W) was larger in common wheat than in spelt (Table 1). Nevertheless, within both sub-sets there were no significant differences in the bread-making quality of the two groups (loaf volume), although the mean value of spelt was 30 cc higher than that of common wheat.

### 3.2. Variability for Grain and Flour Quality Traits in Spelt

**Figure 1:**
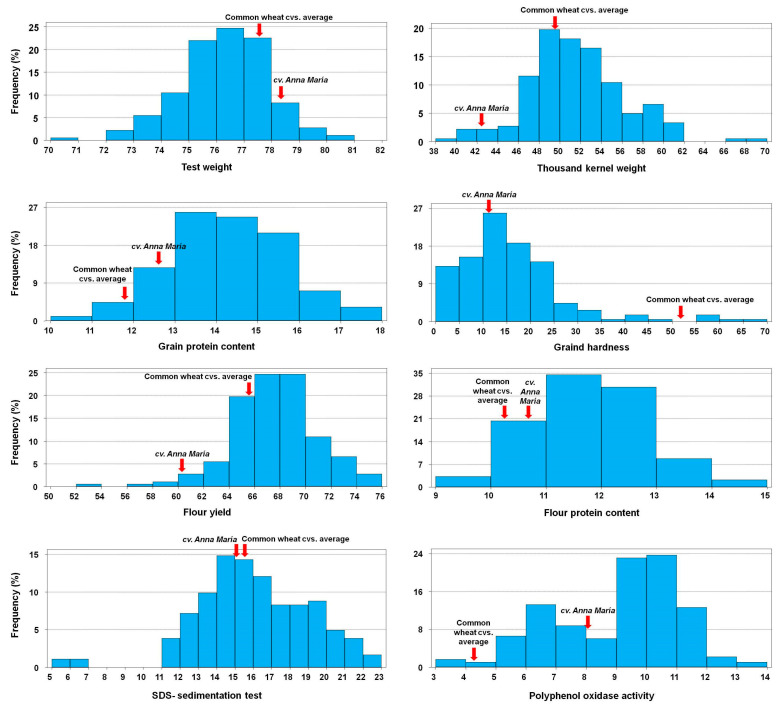
Frequency distribution of the spelt genotypes and average values of the common wheat cultivars for different grain and flour quality traits.

When the comparison was carried out among the spelt genotypes (Figure 1), both traditional and modern spelt (cv. Anna Maria), the data showed high variation among these genotypes for all measured traits in grain and flour (Table S2). The ANOVA analysis suggested a high influence of the genotype in this variation (Table S4), although the differences between both years were also significant.

Most of the traditional spelt genotypes showed lower TW values than the cv. Anna Maria; however, the thousand kernel weight (TKW) of the latter was significantly lower than the values of the traditional spelt accessions (Figure 1).

The protein content, both in grain and flour, was highly variable, with cv. Anna Maria being in the low part of the distribution in both cases (Table S2). This high protein content had little effect on the grain hardness, because, in general, the spelt genotypes showed soft or very soft grain, although some accessions showed values of semi-hard grain (Figure 1). The general lower grain hardness associated with the spelt accessions was positively associated with flour yield.

The gluten strength measured as the SDS-sedimentation volume showed values of medium and high, with some exceptions (Figure 1).

For the PPO activity, the range was very wide (3–14 Ug^−1^min^−1^), and two groups of materials could be distinguished among the spelt genotypes: one with a mean value of 6.5 Ug^−1^min^−1^, and another with the mean values around 10.5 Ug^−1^min^−1^.

### 3.3. Alveogram and Baking Traits in the Spelt Collection

In the previous comparison with common wheat (Table 1), the data showed that spelt doughs had low tenacity, high extensibility, and low to medium gluten strength, as indicated by their W values. The analysis of the 80 genotypes that could be evaluated with the alveograph, showed a wide variation for the traits measured with this equipment (Table 2 and Table S3), with different genotypes exhibiting values higher or lower than the average. In this respect, some spelt genotypes could be classified as medium to high gluten strength with W up to 388 × 10^−4^ J. Spelt genotype BGE 020900 (W = 320 × 10^−4^ J on average across the two years) could be considered a good donor of this trait for breeding programs. For gluten extensibility, several spelt accessions (such as BGE 001990, PI 348727, and PI 348747) showed very low P/L values (0.3), and could be considered interesting sources of this trait. Accession PI 348465 showed a very interesting combination of both gluten strength and extensibility (W = 283 × 10^−4^ J and P/L = 0.4) and could be considered the best material found in terms of gluten quality. The modern spelt (cv. Anna Maria) presented values around the average values of the spelt set (Table 2). In this case, the ANOVA analysis also showed the high influence of the genotype in the variation detected (Table S4).

**Figure 2:**
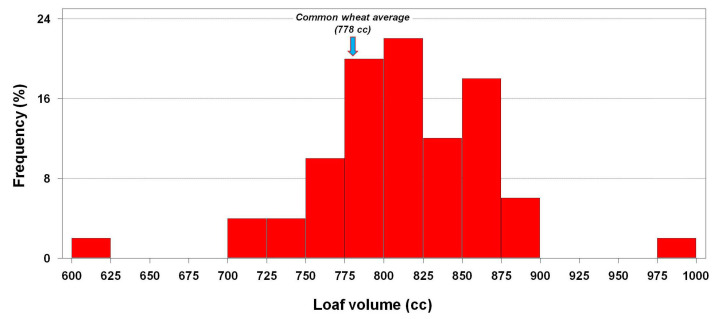
Frequency distribution of the spelt genotypes and average values of the common wheat cultivars for loaf volume.

As already mentioned, the mean values of spelt and common wheat for loaf volume did not show significant differences (Table 1). However, when the 50 genotypes of the spelt set were independently analysed, these materials exhibited a high variability for this trait, with minimum and maximum values of 600 and 975 cc, respectively (Figure 2). Apart from that, almost 82% of these genotypes had loaf volume between 750 and 875 cc. Genotypes PI 469058 and PI 469051 (885 mL and 848 mL of loaf volume, respectively, on average across the two years) were the best performers for this trait and could be used by breeding programs aimed at the improvement of bread-making quality.

Finally, a correlation analysis was carried out with the analyzed traits (except loaf volume, due to the lack of this data in many genotypes) within the spelt wheat set (Table S5). A negative correlation was found between test weight and protein content, and grain size (TKW) and alveograph W. Positive correlations were identified between protein content and SDS-sed and gluten extensibility (L), and among the different alveograph parameters.

## 4. Discussion

Recent changes in the agri-food industry have generated a growing interest in foods and old crops that have been practically lost during the last century [44]. In some cases, this renaissance has been associated with the supposed miraculous properties of these old crops. In general, such statements are not supported by any scientific basis and both the nutritional and nutraceutical properties of ancient wheats have been shown to be very similar to those of modern wheat [45,46,47,48,49,50,51]. However, other real benefits, such as the expansion of diversity in food, are little appreciated.

Within the wheat world, both the old varieties that had been replaced by more productive ones, and some of the species that were cultivated in the past have been recovered during recent decades [6,45]. Some of the latter are called “ancient wheats” and consists mainly of three species: einkorn (*T. monococcum* L. ssp. *monococcum*, 2n = 2× = 14, A^m^A^m^), emmer, and spelt. Some of the agronomic characteristics of these ancient wheats were those that led to their disappearance and abandonment when most of the agricultural processes were mechanized. In addition, due to being hulled grain cereals, the need for special dehulling treatment prior to grinding increased costs and affected their profitability. For this reason, their revival is linked to the boom in traditional and gourmet bakeries, where the higher prices of these products can offset production costs. Nowadays larger retailers also offer flour and products made from these types of wheat.

At the same time, this renewed interest has resulted in the development of numerous studies on these species, comparing their characteristics with the ones of modern wheat [50,51]. However, many of these studies have been carried out with a limited number of genotypes [37], which could create bias in the results and undervalue the true role of these old materials. For this reason, the evaluation of large collections of these ancient wheats, with the limitation of the storage materials in germplasm banks, could shed light on these questions, identifying new genotypes with potential utility for breeding programs. In the current study, one collection of 88 Spanish traditional spelt accessions, together with 9 common wheat cultivars and 1 modern spelt cultivar (cv. Anna Maria), were analyzed and compared for several traits related to milling, processing, and end-use quality.

When the analyzed materials are ancient or old wheats, the technological quality must be evaluated with caution. Changes in baking techniques throughout the last century have generated materials adapted to these techniques, which are different from those required for traditional baking, and consequently, the evaluation of ancient wheats according to modern parameters could not be the best strategy. In this regard, our previous studies on the grain composition of the spelt accessions evaluated in the current study showed the presence of rare HMWGs variants in spelt wheat (1, 13 + 16, and 2 + 12) [38,39]. However, it is possible that the high frequency of these variants is an empirical consequence of the way these wheats were and are used in traditional agri-food applications. These characteristics are mainly demanded by bakers, since all the studies carried out suggest the clear influence of glutenins on the viscoelastic properties of wheat dough [52]. However, millers are interested more in other traits more related to the physical and chemical characteristics of the grain such as TW, TKW, protein content, and grain hardness, mainly due to their influence on the flour yield.

Previous studies have shown that the grain size in spelt is larger for the modern material (with common wheat introgression) than for the traditional spelt [9,31,33]. In this study, the traditional spelt genotypes presented a TW and TKW very similar to the common wheat cultivars used as the control, and, compared with the modern spelt (cv. Anna Maria), the latter has a better TW but its TKW was lower. This reinforces the idea that variation in the traditional spelt is high and could be interesting for breeding programs aiming to develop new cultivars with very high grain size [53,54]. In parallel, the spelt genotypes with high grain size showed high protein content (indeed, a significant correlation between TKW and protein content was found) and a soft texture, which positively affected flour yield.

The PPO activity appeared comparatively higher in spelt than in common wheat. This trait is regulated by several enzymes, synthesized by the *Ppo-1* and *Ppo-2* loci at the homoeologous group 2 chromosomes [55,56,57,58,59], and has been associated with the discoloration and darkening of wheat products [60,61,62,63], which generates a certain amount of rejection among consumers [41,56,61,62,63,64]. Paradoxically, it may happen that today’s consumers associate this dark color with the true presence of flour spelt in a food product, while the cream color suggests that the product is made with flour of modern common wheat and not from spelt (more “natural” vs. more “industrial”). Therefore, high PPO activity may not be an undesirable trait for spelt cultivars, although it does deserve attention if spelt is to be used in the breeding of modern common wheat as source of other traits of interest. In this regard, some traditional spelt genotypes showed low PPO activity values (≤5 Ug^−1^min^−1^) (Table S2), although this was not the general trend.

Previous studies conducted on spelt revealed that spelt mostly exhibits low to medium gluten strength [28,29,30,31,32,33,34]; however, our study revealed that it is also possible to identify genotypes with stronger gluten. In any case, the viscoelastic properties of spelt could be different from those of common wheat. The spelt genotypes showed, in general, more extensible doughs, which was favored by higher protein contents, and, in a few cases, the W values were reasonably high (up to 300 × 10^−4^ J). This was also due to the strong correlation found between alveograph W and P/L, which is also normal in common wheat sets [65]. However, it was not possible to identify an unambiguous relationship between the W values and loaf volume. As with other measured traits, the variation of these two parameters was high among the spelt genotypes. When the loaf volume was related to the flour protein content, some genotypes with low protein content showed high loaf volume, which suggests the high quality of these gluten proteins.

The current trend in the cereal’s world has extended the search for other desirable traits within the grain components, mainly related with nutritional and nutraceutical properties, which would complement the technological properties of the doughs [37,66,67,68]. Nowadays, the presence of micronutrients such as Fe or Zn in the flour, or dietary fiber in form of soluble arabinoxylans, is highly recommended and this has increased the interest in the ancient wheats, with some studies suggesting that these old materials could be a good source for these traits [69,70,71]. In this respect, a previous study on these nutritional aspects has revealed that the current spelt genotypes show a notable variation in these traits [72]. These data, together with the data obtained in the current study, highlight the need to increase the evaluation of wide collections of these ancient wheats, in order to detect the true variability in these old materials for different traits, including those associated with processing and nutritional quality. Such analyses will allow the identification of unique germplasms that could be used for the selection and purification of intra-accession variability for the development of traditional and homogeneous spelt varieties, both to be crossed with modern wheat to transfer the trait of interest, to improve modern wheat genetic diversity, and to develop better-adapted spelt cultivars.

## 5. Conclusions

Ancient wheats can be good sources of interesting agronomic features, mainly rust-resistant genes and quality traits for wheat breeding. The evaluation of the large collections of these old materials would allow for evaluation of the true variability present in these species. In the current study, large variation was found in a set of Spanish spelt landraces, which, in general, showed soft grain, medium–high protein content, low gluten strength, high gluten extensibility, and medium bread-making quality; spelt genotypes showing outstanding values for some of these traits that could be useful for breeding purposes were identified. Additionally, this and similar studies could provide the opportunity to develop new cultivars of spelt with good characteristics for the food industry.

## Figures and Tables

**Table 1:** Average values of the common wheat and spelt groups (averaging genotypes and years) and result of the *t*-test done between both values.

Trait	No. Genotypes (Common: Spelt)	Common Wheat (Mean ± s.d.)	Spelt (Mean ± s.d.)	t-Value
Grain/flour components				
TW (kg/hL)	9:89	77.60 ± 2.77	76.26 ± 1.84	3.94 ***
TKW (g)	9:89	49.73 ± 6.03	51.72 ± 4.81	−2.30 *
GPC (%)	9:89	11.87 ± 1.14	14.27 ± 1.76	−8.01 ***
Hardness (%)	9:89	52.44 ± 15.07	15.78 ± 11.02	18.32 ***
Flour yield (%)	9:89	65.78 ± 6.06	67.53 ± 3.27	−2.78 **
FPC (%)	9:89	10.22 ± 0.72	11.82 ± 0.98	−9.53 ***
SDS-sed (mL)	9:89	15.22 ± 2.65	15.86 ± 3.03	−1.22 ns
PPO activity (Ug^−1^min^−1^)	9:89	4.25 ± 1.75	9.08 ± 2.06	−13.54 ***
Alveogram parameters				
P (mm)	7:80	141.57 ± 26.18	59.42 ± 16.48	16.93 ***
L (mm)	7:80	84.93 ± 18.13	123.83 ± 23.20	−6.10 ***
P/L (ratio)	7:80	1.79 ± 0.68	0.51 ± 0.24	15.27 ***
P/G (ratio)	7:80	7.08 ± 1.96	2.47 ± 0.89	16.33 ***
W (×10^−4^ J)	7:80	437.71 ± 117.33	186.56 ± 57.04	14.16 ***
Ie (%)	7:80	61.33 ± 10.35	46.10 ± 6.45	8.00 ***
Loaf parameters				
Loaf Volume (cc)	4:50	778.00 ± 28.10	809.50 ± 57.64	−1.04 ns

TW, test weight; TKW, thousand kernel weight; GPC, grain protein content; FPC, flour protein contents; SDS-sed, sodium dodecyl sulfate sedimentation test; PPO activity, polyphenol oxidase activity; P, dough tenacity; L, dough extensibility; G: swelling index; W, dough strength; and Ie, elasticity index. ***, **, *: significant at 99.9, 99, and 95%; ns: not significant.

**Table 2:** Comparison of the alveograph parameters obtained in the traditional spelt accessions and the modern spelt cultivar Anna Maria.

Trait	Traditional Spelt		cv. Anna Maria
	Mean ± s.d.	Range	Mean ± s.d.
P (mm)	59.48 ± 16.58	29.00–138.00	55.00 ± 4.24
L (mm)	123.87 ± 23.31	55.00–186.00	120.50 ± 17.67
P/L (ratio)	0.51 ± 024	0.20–1.70	0.45 ± 0.02
P/G (ratio)	2.47 ± 0.90	1.00–6.70	2.30 ± 0.00
W (×10^−4^ J)	186.49 ± 57.18	73.00–388.00	192.00 ± 62.22
Ie (%)	46.04 ± 6.42	28.40–63.20	51.20 ± 10.32

P, dough tenacity; L, dough extensibility; G: swelling index; W, dough strength; and Ie, elasticity index.

## Data Availability

Data are included in the Supplementary Materials.

## Supplementary Materials

The following supporting information can be downloaded at: https://www.mdpi.com/article/10.3390/foods12162996/s1, Table S1: Plant material used in the study; Table S2: Mean values of the grain and flour measured traits for each season in the materials evaluated (spelt and common wheat); Table S3: Mean values of the alveographic and baking traits for each season in the materials evaluated (spelt and common wheat); Table S4: Effects of genotype, season, and genotype x season (GxS) on quality traits in spelt accessions. Sum of squares, % of the total sum of squares from ANOVA analysis, and coefficient of variation (CV) are indicated; and Table S5: Correlation analysis of quality traits in spelt.
