# Supplementary material for: Processing and Bread-Making Quality Profile of Spanish Spelt Wheat
Source: Foods. 2023 Aug 9;12(16):2996. doi: 10.3390/foods12162996 (PMC10453461; doi:10.3390/foods12162996)
Supplement: Supplementary file 1 [file foods-12-02996-s001.zip › foods-2520763-supplementary.pdf]

Supplementary materials

Table S1. Plant material used in the study.

Germplasm Bank	**Accession Number**
Triticum aestivum ssp. *spelta* (spelt)*	
Centro de Recursos Fitogeneticos (INIA, Spain)<br><br>- 26 accessions -	BGE 012902; BGE 012903; BGE 012906; BGE 001947; BGE 001972; BGE 001978; BGE 001990; BGE 002002; BGE 002005; BGE 002006; BGE 012766; BGE 012911; BGE 012920; BGE 012931; BGE 012932; BGE 012935; BGE 012937; BGE 014252; BGE 014270; BGE 017153; BGE 020898; BGE 020900; BGE 020903; BGE 020935; BGE 023734; BGE 025420
National Small Grains Collection (USDA, USA)<br><br>- 62 accessions -	PI 348428; PI 348439; PI 348455; PI 348458; PI 348462; PI 348463; PI 348465; PI 348471; PI 348473; PI 348478; PI 348480; PI 348483; PI 348489; PI 348493; PI 348495; PI 348515; Pi 348519; PI 348544; PI 348570; PI 348572; PI 348676; PI 348693; PI 348696; PI 348698; PI 348701; PI 348702; PI 348712; PI 348727; PI 348728; PI 348741; PI 348747; PI 348767; PI 348771; PI 469022; PI 469023; PI 469024; PI 469026; PI 469028; PI 469029; PI 469030; PI 469031; PI 469032; PI 469034; PI 469038; PI 469039; PI 469040; PI 469041; PI 469042; PI 469045; PI 469046; PI 469047; PI 469048; PI 469049; PI 469050; PI 469051; PI 469053; PI 469054; PI 469056; PI 469057; PI 469058; PI 469059; PI 469060
Triticum aestivum ssp. *spelta* (modern spelt)	
cv. Anna Maria	
Triticum aestivum ssp. *aestivum* (common wheat)	
cv. Antequera	
cv. Arthur Nick	
cv. Conil	
cv. Galera	
cv. Montemayor	
cv. Rota	
cv. Santaella	
cv. Setenil	
cv. Tejada	

*All the spelt accessions have Spanish origin

Table S2. Mean values of the grain and flour measured traits for each season in the materials evaluated (spelt and common wheat).

Genotype	Season	Type	TW (Kg/Hl)	TKW (g)	GPC (%)	HARDNESS (%)	FLOUR YIELD (%)	FPC (%)	SDS-sed. (ml)	PPO Activity ($Ug^{-1}min^{-1}$)
BGE 020900	2019-2020	traditional spelt	77.25	43.14	14.38	62.64	69.83	12.30	16.00	8.38
BGE 020900	2020-2021	traditional spelt	80.89	48.66	13.77	68.06	65.98	11.23	15.50	5.66
BGE 020903	2019-2020	traditional spelt	77.95	40.52	14.33	34.35	65.43	11.75	11.50	6.78
BGE 020903	2020-2021	traditional spelt	77.85	49.98	15.59	23.02	60.25	12.02	14.00	5.11
BGE 001947	2019-2020	traditional spelt	77.35	51.96	12.11	14.47	65.75	11.24	14.50	10.66
BGE 001947	2020-2021	traditional spelt	75.99	56.55	12.35	8.54	64.27	10.59	15.00	9.02
BGE 001972	2019-2020	traditional spelt	76.10	53.04	13.15	11.46	69.46	11.29	13.50	10.46
BGE 001972	2020-2021	traditional spelt	74.13	59.37	13.24	4.27	65.09	10.26	12.00	10.11
BGE 001978	2019-2020	traditional spelt	75.45	53.86	14.77	12.91	68.77	11.91	15.00	9.96
BGE 001978	2020-2021	traditional spelt	75.05	58.39	15.37	14.12	65.24	12.41	19.50	9.25
BGE 001990	2019-2020	traditional spelt	77.55	46.73	13.80	8.99	72.57	12.73	17.00	11.50
BGE 001990	2020-2021	traditional spelt	75.27	50.42	14.26	3.33	68.66	12.29	19.00	9.24
BGE 002002	2019-2020	traditional spelt	76.45	50.94	15.39	17.67	67.51	12.69	15.00	10.69
BGE 002002	2020-2021	traditional spelt	73.91	48.06	15.19	10.87	63.39	12.10	16.50	11.04
BGE 002005	2019-2020	traditional spelt	76.25	53.16	13.53	38.79	71.44	11.77	13.00	9.05
BGE 002005	2020-2021	traditional spelt	75.84	55.43	14.86	47.53	70.22	11.69	14.50	9.40
BGE 002006	2019-2020	traditional spelt	76.90	57.46	13.83	20.76	67.68	12.89	16.00	6.42
BGE 002006	2020-2021	traditional spelt	75.46	59.91	14.42	14.00	65.86	11.41	15.00	6.39
BGE 012766	2019-2020	traditional spelt	80.15	39.47	11.85	0.79	65.06	10.07	14.00	13.29
BGE 012766	2020-2021	traditional spelt	77.18	40.87	13.75	1.00	57.66	10.42	16.00	12.88
BGE 012902	2019-2020	traditional spelt	77.55	50.18	15.16	14.85	65.08	12.47	17.50	7.12
BGE 012902	2020-2021	traditional spelt	75.92	53.05	16.47	8.93	61.83	12.41	20.00	7.46
BGE 012903	2019-2020	traditional spelt	76.10	48.80	14.47	2.81	64.39	11.97	15.50	10.39
BGE 012903	2020-2021	traditional spelt	72.73	50.78	14.02	0.76	62.59	11.40	16.50	10.40
BGE 012906	2019-2020	traditional spelt	74.05	50.22	13.79	29.55	70.50	11.43	13.50	6.90
BGE 012906	2020-2021	traditional spelt	77.11	53.75	14.68	24.32	64.80	12.10	16.50	6.81

Genotype	Season	Type	TW (Kg/Hl)	TKW (g)	GPC (%)	HARDNESS (%)	FLOUR YIELD (%)	FPC (%)	SDS-sed. (ml)	PPO Activity ($Ug^{-1}min^{-1}$)
BGE 012911	2019-2020	traditional spelt	77.35	52.69	15.44	16.68	74.14	13.55	20.00	9.14
BGE 012911	2020-2021	traditional spelt	75.09	54.63	15.41	8.64	69.33	12.79	21.50	9.36
BGE 012920	2019-2020	traditional spelt	75.00	58.60	13.47	16.72	67.35	11.42	13.00	10.47
BGE 012920	2020-2021	traditional spelt	75.05	61.29	14.45	17.92	63.27	10.98	14.50	9.57
BGE 012931	2019-2020	traditional spelt	75.80	52.03	12.48	17.10	67.36	11.42	14.00	9.31
BGE 012931	2020-2021	traditional spelt	76.18	54.80	12.58	7.79	61.72	10.47	14.00	9.34
BGE 012932	2019-2020	traditional spelt	76.85	51.87	15.16	10.46	65.88	12.44	16.00	10.62
BGE 012932	2020-2021	traditional spelt	74.73	50.69	13.81	2.35	64.25	11.23	15.50	10.90
BGE 012935	2019-2020	traditional spelt	78.75	53.11	13.03	17.90	70.93	11.42	15.00	5.71
BGE 012935	2020-2021	traditional spelt	76.96	53.36	14.14	11.95	68.52	11.45	16.50	5.53
BGE 012937	2019-2020	traditional spelt	75.90	47.92	14.02	21.79	65.59	11.70	12.50	11.71
BGE 012937	2020-2021	traditional spelt	74.76	52.24	13.46	7.62	65.97	10.64	13.50	11.49
BGE 014252	2019-2020	traditional spelt	74.85	49.92	17.12	21.85	66.02	14.51	16.00	11.87
BGE 014252	2020-2021	traditional spelt	76.57	51.24	15.15	19.11	65.20	12.23	15.00	11.60
BGE 014270	2019-2020	traditional spelt	73.30	67.00	13.85	44.82	73.94	11.53	6.00	3.78
BGE 014270	2020-2021	traditional spelt	73.12	69.38	13.23	43.27	71.03	10.84	5.50	3.46
BGE 017153	2019-2020	traditional spelt	77.80	48.78	11.39	22.50	68.49	10.28	13.00	10.57
BGE 017153	2020-2021	traditional spelt	77.06	51.72	13.31	18.62	65.45	10.98	15.00	9.58
BGE 020898	2019-2020	traditional spelt	77.35	42.75	11.27	16.16	64.72	9.59	11.00	6.73
BGE 020898	2020-2021	traditional spelt	77.57	46.42	12.82	7.69	64.96	10.27	13.50	6.07
BGE 020935	2019-2020	traditional spelt	75.95	52.03	14.79	23.37	68.80	12.51	14.50	11.06
BGE 020935	2020-2021	traditional spelt	73.43	53.86	14.94	16.51	67.20	12.31	17.00	10.88
BGE 023734	2019-2020	traditional spelt	75.45	51.78	14.87	21.40	63.72	12.64	13.50	7.31
BGE 023734	2020-2021	traditional spelt	75.46	51.21	14.90	16.16	61.61	11.54	14.00	7.24
BGE 025420	2019-2020	traditional spelt	78.90	44.14	12.07	22.74	65.43	10.75	12.50	12.14
BGE 025420	2020-2021	traditional spelt	78.33	47.83	13.50	14.15	64.72	10.91	14.50	11.67
PI 348428	2019-2020	traditional spelt	77.70	53.00	13.89	2.18	69.21	11.76	15.00	10.34
PI 348428	2020-2021	traditional spelt	76.78	55.26	16.31	2.74	65.19	12.58	18.00	9.85

Genotype	Season	Type	TW (Kg/Hl)	TKW (g)	GPC (%)	HARDNESS (%)	FLOUR YIELD (%)	FPC (%)	SDS-sed. (ml)	PPO Activity ($Ug^{-1}min^{-1}$)
PI 348439	2019-2020	traditional spelt	75.95	48.61	14.84	15.50	72.34	13.22	18.50	6.21
PI 348439	2020-2021	traditional spelt	75.20	52.20	14.25	13.65	69.17	12.12	18.50	8.08
PI 348455	2019-2020	traditional spelt	76.25	47.63	12.99	20.62	71.07	12.29	14.00	11.17
PI 348455	2020-2021	traditional spelt	76.01	48.61	12.43	7.05	67.87	10.85	14.50	10.18
PI 348458	2019-2020	traditional spelt	78.00	48.92	15.16	11.18	69.31	12.95	15.50	9.34
PI 348458	2020-2021	traditional spelt	76.14	49.50	15.53	6.14	69.87	12.51	19.00	7.72
PI 348462	2019-2020	traditional spelt	76.40	51.50	11.77	12.25	69.75	11.20	14.00	10.74
PI 348462	2020-2021	traditional spelt	73.72	53.18	15.20	2.21	67.48	12.05	17.00	9.45
PI 348463	2019-2020	traditional spelt	76.10	46.91	12.58	13.87	67.68	10.62	12.50	10.38
PI 348463	2020-2021	traditional spelt	75.47	49.15	14.00	6.30	67.48	10.90	14.00	9.79
PI 348465	2019-2020	traditional spelt	78.45	46.73	15.30	12.56	68.04	12.36	18.00	10.92
PI 348465	2020-2021	traditional spelt	76.21	49.90	14.59	5.02	69.82	11.56	20.50	10.10
PI 348471	2019-2020	traditional spelt	77.10	46.33	12.30	20.96	69.62	10.53	13.50	10.05
PI 348471	2020-2021	traditional spelt	76.53	47.51	13.10	17.11	68.04	10.42	14.50	9.80
PI 348473	2019-2020	traditional spelt	79.25	45.47	13.84	15.23	65.70	11.02	12.50	9.98
PI 348473	2020-2021	traditional spelt	78.37	47.37	15.10	15.96	68.30	12.17	19.50	9.33
PI 348478	2019-2020	traditional spelt	77.65	50.16	16.31	17.02	69.37	12.38	16.00	9.79
PI 348478	2020-2021	traditional spelt	76.77	49.39	13.97	14.10	70.04	11.95	18.00	9.79
PI 348480	2019-2020	traditional spelt	75.95	48.77	13.46	28.84	69.61	11.41	16.00	10.67
PI 348480	2020-2021	traditional spelt	74.62	49.86	15.63	16.34	67.57	12.27	20.00	10.50
PI 348483	2019-2020	traditional spelt	78.45	47.29	14.04	19.80	69.77	11.62	15.00	9.51
PI 348483	2020-2021	traditional spelt	77.32	50.85	14.53	16.69	66.77	11.70	19.00	8.38
PI 348489	2019-2020	traditional spelt	79.05	54.99	14.71	13.73	67.34	12.38	16.50	11.29
PI 348489	2020-2021	traditional spelt	76.22	55.09	14.35	10.59	68.31	11.71	15.00	10.73
PI 348493	2019-2020	traditional spelt	75.75	48.98	17.14	13.74	66.85	14.22	21.00	9.86
PI 348493	2020-2021	traditional spelt	73.75	49.08	17.28	3.95	66.94	13.52	21.00	10.10
PI 348495	2019-2020	traditional spelt	77.20	44.55	11.65	27.51	71.75	10.84	12.50	11.07
PI 348495	2020-2021	traditional spelt	77.43	48.11	12.76	16.47	65.47	10.85	14.50	10.23

Genotype	Season	Type	TW (Kg/Hl)	TKW (g)	GPC (%)	HARDNESS (%)	FLOUR YIELD (%)	FPC (%)	SDS-sed. (ml)	PPO Activity ($Ug^{-1}min^{-1}$)
PI 348515	2019-2020	traditional spelt	77.50	46.26	13.71	21.06	72.45	11.63	14.00	6.45
PI 348515	2020-2021	traditional spelt	75.07	46.00	15.05	15.92	61.13	11.94	18.00	7.06
PI 348519	2019-2020	traditional spelt	78.75	51.08	11.65	19.48	68.41	10.34	11.50	11.10
PI 348519	2020-2021	traditional spelt	75.51	53.99	13.33	6.42	68.53	10.87	15.00	9.30
PI 348544	2019-2020	traditional spelt	74.00	49.90	17.20	19.26	67.83	14.10	21.00	6.55
PI 348544	2020-2021	traditional spelt	76.38	54.41	13.98	9.21	67.57	11.59	18.00	7.17
PI 348570	2019-2020	traditional spelt	77.40	47.75	12.92	14.36	65.68	11.39	14.00	8.78
PI 348570	2020-2021	traditional spelt	75.30	50.71	15.32	5.92	65.92	12.29	18.00	9.06
PI 348572	2019-2020	traditional spelt	75.95	50.46	12.39	27.48	68.24	10.87	13.50	5.74
PI 348572	2020-2021	traditional spelt	73.06	50.50	13.78	19.27	66.41	11.23	18.50	5.31
PI 348676	2019-2020	traditional spelt	76.70	47.33	15.12	31.06	71.10	12.33	16.00	7.59
PI 348676	2020-2021	traditional spelt	75.27	47.53	15.63	26.25	64.75	11.96	19.50	6.71
PI 348693	2019-2020	traditional spelt	76.90	48.80	13.07	24.35	67.27	11.57	13.50	11.06
PI 348693	2020-2021	traditional spelt	74.11	49.26	16.76	13.85	64.90	13.54	20.00	11.57
PI 348696	2019-2020	traditional spelt	78.30	47.88	15.00	23.33	67.24	12.36	17.00	10.40
PI 348696	2020-2021	traditional spelt	76.78	47.37	15.16	25.43	73.47	11.88	17.50	9.44
PI 348698	2019-2020	traditional spelt	77.05	49.99	14.55	19.36	67.04	12.61	16.00	9.49
PI 348698	2020-2021	traditional spelt	73.30	52.18	16.15	15.60	66.15	12.68	16.50	10.02
PI 348701	2019-2020	traditional spelt	76.25	48.68	13.46	16.23	70.27	11.24	13.00	7.54
PI 348701	2020-2021	traditional spelt	76.82	50.86	13.85	2.71	65.80	11.43	14.00	7.94
PI 348702	2019-2020	traditional spelt	72.65	51.65	15.14	14.95	66.65	11.95	15.00	7.58
PI 348702	2020-2021	traditional spelt	70.58	52.83	16.09	11.63	63.63	13.08	21.00	6.07
PI 348712	2019-2020	traditional spelt	76.10	50.17	12.25	26.66	69.94	11.03	15.00	6.25
PI 348712	2020-2021	traditional spelt	78.18	51.58	13.99	14.50	69.99	11.59	18.00	6.34
PI 348727	2019-2020	traditional spelt	76.90	48.99	13.67	14.18	72.46	11.63	14.00	9.38
PI 348727	2020-2021	traditional spelt	76.95	48.25	12.61	13.06	68.25	10.44	12.00	9.39
PI 348728	2019-2020	traditional spelt	77.80	46.92	15.12	24.07	70.81	12.68	19.00	5.26
PI 348728	2020-2021	traditional spelt	75.55	50.30	15.25	15.62	72.58	12.03	20.00	3.72

Genotype	Season	Type	TW (Kg/Hl)	TKW (g)	GPC (%)	HARDNESS (%)	FLOUR YIELD (%)	FPC (%)	SDS-sed. (ml)	PPO Activity ($Ug^{-1}min^{-1}$)
PI 348741	2019-2020	traditional spelt	76.25	48.35	13.73	24.00	64.61	11.40	15.00	5.97
PI 348741	2020-2021	traditional spelt	74.95	52.52	15.18	14.46	63.65	11.83	17.50	6.51
PI 348747	2019-2020	traditional spelt	77.35	49.10	16.08	21.05	70.60	13.34	17.00	8.74
PI 348747	2020-2021	traditional spelt	75.58	52.97	16.57	10.94	67.02	13.43	20.50	10.35
PI 348767	2019-2020	traditional spelt	76.90	52.18	13.49	17.01	66.56	11.87	17.50	10.07
PI 348767	2020-2021	traditional spelt	75.06	55.77	15.32	9.00	69.20	13.08	22.00	10.00
PI 348771	2019-2020	traditional spelt	77.20	49.34	15.45	9.09	63.06	13.40	19.00	9.04
PI 348771	2020-2021	traditional spelt	74.32	49.44	15.14	3.49	65.48	12.30	19.50	9.66
PI 469022	2019-2020	traditional spelt	77.35	56.20	15.95	22.91	74.14	13.25	16.00	9.81
PI 469022	2020-2021	traditional spelt	74.82	56.89	14.76	11.83	71.43	12.27	22.00	10.34
PI 469023	2019-2020	traditional spelt	74.25	56.41	15.65	13.91	68.54	13.22	20.00	7.59
PI 469023	2020-2021	traditional spelt	75.14	56.28	14.20	13.61	68.50	11.34	16.00	7.33
PI 469024	2019-2020	traditional spelt	75.90	50.96	15.70	23.84	71.26	13.01	18.00	6.61
PI 469024	2020-2021	traditional spelt	73.19	52.46	16.36	13.90	68.47	13.54	21.00	6.12
PI 469026	2019-2020	traditional spelt	77.35	50.11	14.28	13.06	72.13	12.29	17.50	10.91
PI 469026	2020-2021	traditional spelt	76.95	52.90	13.12	11.46	66.88	10.54	18.00	10.41
PI 469028	2019-2020	traditional spelt	77.20	52.81	14.35	23.15	67.08	12.02	16.50	5.78
PI 469028	2020-2021	traditional spelt	76.02	54.75	13.40	15.64	66.69	10.96	15.50	6.97
PI 469029	2019-2020	traditional spelt	77.95	48.64	11.53	9.25	72.56	10.40	11.00	8.93
PI 469029	2020-2021	traditional spelt	78.35	49.08	12.31	3.28	67.43	10.19	12.00	8.67
PI 469030	2019-2020	traditional spelt	76.60	48.44	14.06	16.25	71.72	12.01	17.50	11.60
PI 469030	2020-2021	traditional spelt	75.28	50.83	17.67	12.72	68.92	13.62	21.00	11.39
PI 469031	2019-2020	traditional spelt	75.15	47.14	15.76	8.03	63.48	12.82	17.50	12.07
PI 469031	2020-2021	traditional spelt	72.15	52.99	15.18	1.00	53.32	12.09	19.50	10.97
PI 469032	2019-2020	traditional spelt	76.85	51.40	11.74	9.33	70.09	10.80	13.00	6.92
PI 469032	2020-2021	traditional spelt	75.43	55.10	12.24	4.18	67.48	9.79	14.00	7.04
PI 469034	2019-2020	traditional spelt	77.20	50.88	12.86	2.75	69.09	10.99	12.50	11.54
PI 469034	2020-2021	traditional spelt	75.72	57.37	12.42	1.00	64.40	9.39	11.00	10.11

Genotype	Season	Type	TW (Kg/Hl)	TKW (g)	GPC (%)	HARDNESS (%)	FLOUR YIELD (%)	FPC (%)	SDS-sed. (ml)	PPO Activity ($Ug^{-1}min^{-1}$)
PI 469038	2019-2020	traditional spelt	73.60	54.64	14.89	56.50	59.33	12.90	6.50	13.55
PI 469038	2020-2021	traditional spelt	72.14	60.42	13.40	42.35	70.16	10.65	5.00	12.60
PI 469039	2019-2020	traditional spelt	77.70	57.30	13.80	7.93	67.64	11.65	15.00	5.16
PI 469039	2020-2021	traditional spelt	75.11	59.62	14.16	4.50	64.30	11.29	15.50	5.03
PI 469040	2019-2020	traditional spelt	77.70	54.34	13.28	22.54	72.67	11.69	14.00	10.71
PI 469040	2020-2021	traditional spelt	76.92	53.46	12.73	14.37	67.74	10.47	13.50	10.57
PI 469041	2019-2020	traditional spelt	77.05	49.05	12.00	18.43	70.65	10.26	13.50	11.19
PI 469041	2020-2021	traditional spelt	77.12	52.04	12.28	9.78	70.35	9.93	13.50	9.97
PI 469042	2019-2020	traditional spelt	76.90	58.59	13.80	25.40	63.40	11.15	12.00	6.95
PI 469042	2020-2021	traditional spelt	75.26	60.25	16.07	18.39	64.15	11.85	14.50	6.01
PI 469045	2019-2020	traditional spelt	78.00	54.20	13.79	10.39	68.94	11.35	14.00	6.60
PI 469045	2020-2021	traditional spelt	75.30	60.50	15.75	2.94	66.49	12.41	19.00	6.17
PI 469046	2019-2020	traditional spelt	77.65	59.80	13.77	8.86	67.53	11.02	12.50	10.73
PI 469046	2020-2021	traditional spelt	75.65	61.60	16.89	4.98	64.28	12.79	19.00	9.68
PI 469047	2019-2020	traditional spelt	77.85	59.58	14.81	11.77	74.19	11.76	15.50	9.31
PI 469047	2020-2021	traditional spelt	74.39	60.59	16.22	8.66	67.50	13.75	22.00	9.50
PI 469048	2019-2020	traditional spelt	76.70	55.74	14.73	16.62	68.24	12.33	14.00	10.14
PI 469048	2020-2021	traditional spelt	74.97	58.49	15.13	10.48	68.78	12.30	17.50	9.26
PI 469049	2019-2020	traditional spelt	77.85	46.14	13.68	31.65	69.52	11.95	16.00	9.44
PI 469049	2020-2021	traditional spelt	74.28	50.44	14.19	22.05	66.66	11.26	16.50	8.99
PI 469050	2019-2020	traditional spelt	78.15	45.66	13.66	21.16	74.79	11.62	14.50	11.72
PI 469050	2020-2021	traditional spelt	74.76	50.06	15.10	13.89	66.62	12.65	18.50	11.50
PI 469051	2019-2020	traditional spelt	77.70	55.15	16.17	12.46	67.71	13.68	19.50	8.93
PI 469051	2020-2021	traditional spelt	76.96	58.67	14.99	7.67	68.40	12.20	18.50	7.89
PI 469053	2019-2020	traditional spelt	79.10	52.89	13.66	23.57	73.55	12.16	14.50	9.50
PI 469053	2020-2021	traditional spelt	77.66	55.04	14.93	8.50	70.50	12.80	19.50	9.65
PI 469054	2019-2020	traditional spelt	76.70	50.15	14.94	13.16	66.47	12.79	20.00	10.56
PI 469054	2020-2021	traditional spelt	77.41	52.85	13.66	4.68	66.98	11.59	18.00	9.97

Genotype	Season	Type	TW (Kg/Hl)	TKW (g)	GPC (%)	HARDNESS (%)	FLOUR YIELD (%)	FPC (%)	SDS-sed. (ml)	PPO Activity ($Ug^{-1}min^{-1}$)
PI 469056	2019-2020	traditional spelt	76.95	49.89	14.62	33.54	68.69	12.79	15.00	10.55
PI 469056	2020-2021	traditional spelt	74.58	54.26	17.54	23.75	68.14	14.43	19.50	9.60
PI 469057	2019-2020	traditional spelt	76.40	54.75	13.18	20.27	67.94	11.25	13.00	10.75
PI 469057	2020-2021	traditional spelt	76.05	57.52	14.36	9.94	64.12	11.54	15.50	10.20
PI 469058	2019-2020	traditional spelt	76.10	49.40	15.24	12.29	68.52	12.70	16.00	10.87
PI 469058	2020-2021	traditional spelt	74.46	48.57	14.14	7.46	67.92	11.25	15.00	11.02
PI 469059	2019-2020	traditional spelt	76.50	52.50	14.76	13.51	68.07	12.48	15.00	11.60
PI 469059	2020-2021	traditional spelt	75.61	55.80	14.46	11.94	69.17	11.84	17.50	11.15
PI 469060	2019-2020	traditional spelt	78.60	58.26	15.39	2.47	72.62	12.92	17.50	5.57
PI 469060	2020-2021	traditional spelt	76.17	58.38	13.93	1.59	69.04	11.41	19.00	6.34
Anna Maria	2019-2020	modern spelt	79.10	42.07	12.09	9.30	62.26	10.59	13.50	8.76
Anna Maria	2020-2021	modern spelt	77.84	43.46	13.74	12.66	58.58	10.84	16.50	7.65
Antequera	2019-2020	common wheat	77.70	47.16	13.80	46.34	70.37	12.05	20.50	5.42
Antequera	2020-2021	common wheat	81.31	47.47	13.31	65.74	63.15	10.93	20.50	7.87
Arthur Nick	2019-2020	common wheat	74.85	39.98	11.36	23.00	54.52	10.54	13.00	4.40
Arthur Nick	2020-2021	common wheat	76.67	40.80	10.75	32.53	50.06	9.79	11.50	6.22
Conil	2019-2020	common wheat	76.95	49.87	11.68	54.97	74.27	11.00	17.50	2.81
Conil	2020-2021	common wheat	80.01	57.67	12.00	69.36	64.40	9.98	18.00	2.94
Galera	2019-2020	common wheat	72.65	40.32	12.04	60.98	67.02	10.47	17.00	3.23
Galera	2020-2021	common wheat	78.54	42.64	12.02	69.83	65.79	10.38	17.50	5.23
Motemayor	2019-2020	common wheat	73.60	50.97	10.52	27.78	63.87	10.08	13.50	5.52
Motemayor	2020-2021	common wheat	80.12	56.68	10.90	42.79	64.10	9.24	12.50	5.28
Rota	2019-2020	common wheat	75.15	47.43	12.59	48.94	75.75	10.05	12.50	1.96
Rota	2020-2021	common wheat	79.74	50.05	11.70	63.30	70.85	9.51	15.00	1.94
Santaella	2019-2020	common wheat	75.35	54.59	12.80	37.30	67.64	10.49	14.00	1.77
Santaella	2020-2021	common wheat	79.82	54.61	11.32	53.22	64.20	9.40	14.00	2.59
Setenil	2019-2020	common wheat	75.95	56.75	11.26	55.41	68.90	10.03	13.50	4.70
Setenil	2020-2021	common wheat	80.11	55.75	11.90	70.48	64.23	9.53	15.50	6.65

Genotype	Season	Type	TW (Kg/Hl)	TKW (g)	GPC (%)	HARDNESS (%)	FLOUR YIELD (%)	FPC (%)	SDS-sed. (ml)	PPO Activity ($Ug^{-1}min^{-1}$)
Tejada	2019-2020	common wheat	76.30	49.81	12.49	51.80	69.68	11.18	13.50	3.25
Tejada	2020-2021	common wheat	81.91	52.60	11.21	69.81	65.16	9.45	14.50	4.82

TW, test weight; TKW, thousand kernel weight; GPC, grain protein content; FPC , flour protein contents; SDS-sed, Sodium dodecyl sulfate sedimentation test; PPO activity, polyphenol oxidase activity.

Table S3. Mean values of the alveographic and baking traits for each season in the materials evaluated (spelt and common wheat).

Genotypes	Season	Type	P (mm)	L (mm)	P/L (ratio)	P/G (ratio)	W (×10^{-4}J)	Ie (%)	Loaf Volume (cc)
BGE 001947	2019-2020	traditional spelt	63	108	0.6	2.7	170	42.6	760
BGE 001947	2020-2021	traditional spelt	62	110	0.6	2.7	181	45.8	
BGE 001978	2019-2020	traditional spelt	61	135	0.5	2.4	199	45.6	850
BGE 001978	2020-2021	traditional spelt	56	163	0.3	2.0	228	51	
BGE 001990	2019-2020	traditional spelt	47	148	0.3	1.7	173	48.4	808
BGE 001990	2020-2021	traditional spelt	44	144	0.3	1.6	157	48	
BGE 002002	2019-2020	traditional spelt	42	144	0.3	1.6	112	34.6	-
BGE 002002	2020-2021	traditional spelt	54	116	0.5	2.3	147	40.6	
BGE 002005	2019-2020	traditional spelt	75	90	0.8	3.6	199	47.1	795
BGE 002005	2020-2021	traditional spelt	130	76	1.7	6.7	304	45.1	
BGE 002006	2019-2020	traditional spelt	53	98	0.5	2.4	128	38.4	810
BGE 002006	2020-2021	traditional spelt	69	112	0.6	2.9	203	46	
BGE 012766	2019-2020	traditional spelt	65	110	0.6	2.8	236	58.4	-
BGE 012766	2020-2021	traditional spelt	82	113	0.7	3.5	323	63.2	
BGE 012902	2019-2020	traditional spelt	66	139	0.5	2.5	239	50.0	-
BGE 012902	2020-2021	traditional spelt	75	134	0.6	2.9	282	53.2	
BGE 012903	2019-2020	traditional spelt	48	132	0.4	1.9	139	40.9	-
BGE 012903	2020-2021	traditional spelt	56	142	0.4	2.1	205	51.5	
BGE 012906	2019-2020	traditional spelt	90	106	0.9	3.9	294	54.8	-
BGE 012906	2020-2021	traditional spelt	138	87	1.6	6.6	388	53.5	
BGE 012911	2019-2020	traditional spelt	45	109	0.4	1.9	127	43.6	975

Genotypes	Season	Type	P (mm)	L (mm)	P/L (ratio)	P/G (ratio)	W (×10⁻⁴J)	Ie (%)	Loaf Volume (cc)
BGE 012911	2020-2021	traditional spelt	46	174	0.3	1.6	191	49.9	
BGE 012920	2019-2020	traditional spelt	47	129	0.4	1.9	131	39.2	795
BGE 012920	2020-2021	traditional spelt	62	114	0.5	2.6	175	42.5	
BGE 012931	2019-2020	traditional spelt	57	126	0.5	2.3	179	45.0	835
BGE 012931	2020-2021	traditional spelt	60	136	0.4	2.3	221	50.9	
BGE 012932	2019-2020	traditional spelt	63	127	0.5	2.5	199	46.0	820
BGE 012932	2020-2021	traditional spelt	76	112	0.7	3.2	227	46.4	
BGE 012935	2019-2020	traditional spelt	65	124	0.5	2.6	194	43.4	783
BGE 012935	2020-2021	traditional spelt	81	114	0.7	3.4	253	49.4	
BGE 012937	2019-2020	traditional spelt	49	110	0.5	2.1	121	37.3	-
BGE 012937	2020-2021	traditional spelt	57	107	0.5	2.5	156	43.1	
BGE 014252	2019-2020	traditional spelt	44	108	0.4	1.9	109	38.1	750
BGE 014252	2020-2021	traditional spelt	54	99	0.6	2.4	145	44.4	
BGE 017153	2019-2020	traditional spelt	91	83	1.1	4.5	230	47.4	855
BGE 017153	2020-2021	traditional spelt	105	88	1.2	5.0	298	53.1	
BGE 020898	2019-2020	traditional spelt	48	117	0.4	2.0	141	43.7	-
BGE 020898	2020-2021	traditional spelt	81	94	0.9	3.8	229	50	
BGE 020900	2019-2020	traditional spelt	100	92	1.1	4.7	311	57.7	777
BGE 020900	2020-2021	traditional spelt	127	78	1.6	6.4	329	51.3	
BGE 020903	2019-2020	traditional spelt	51	87	0.6	2.5	121	41.3	-
BGE 020903	2020-2021	traditional spelt	56	107	0.5	2.4	151	42.3	
BGE 020935	2019-2020	traditional spelt	57	132	0.4	2.2	207	52.1	823
BGE 020935	2020-2021	traditional spelt	67	133	0.5	2.6	261	55.6	

Genotypes	Season	Type	P (mm)	L (mm)	P/L (ratio)	P/G (ratio)	W ($\times 10^{-4}$J)	Ie (%)	Loaf Volume (cc)
BGE 025420	2019-2020	traditional spelt	52	130	0.4	2.0	156	42.0	-
BGE 025420	2020-2021	traditional spelt	56	127	0.4	2.2	188	48.5	
PI 348428	2019-2020	traditional spelt	52	119	0.4	2.1	142	39.8	795
PI 348428	2020-2021	traditional spelt	54	143	0.4	2.0	183	45.9	
PI 348439	2019-2020	traditional spelt	61	142	0.4	2.3	237	53.3	820
PI 348439	2020-2021	traditional spelt	74	121	0.6	3.0	254	52	
PI 348455	2019-2020	traditional spelt	41	86	0.5	2.0	88	35.9	-
PI 348455	2020-2021	traditional spelt	44	123	0.4	1.8	121	39.4	
PI 348458	2019-2020	traditional spelt	45	109	0.4	1.9	118	40.2	805
PI 348458	2020-2021	traditional spelt	44	138	0.3	1.7	147	46.8	
PI 348462	2019-2020	traditional spelt	53	121	0.4	2.2	169	47.4	855
PI 348462	2020-2021	traditional spelt	43	156	0.3	1.5	176	53.4	
PI 348463	2019-2020	traditional spelt	51	123	0.4	2.1	165	48.0	805
PI 348463	2020-2021	traditional spelt	47	142	0.3	1.8	189	55.1	
PI 348465	2019-2020	traditional spelt	64	151	0.4	2.3	276	56.2	-
PI 348465	2020-2021	traditional spelt	67	144	0.5	2.5	290	58.7	
PI 348471	2019-2020	traditional spelt	60	112	0.5	2.5	197	52.1	855
PI 348471	2020-2021	traditional spelt	58	125	0.5	2.3	208	53.5	
PI 348473	2019-2020	traditional spelt	45	160	0.3	1.6	165	46.4	840
PI 348473	2020-2021	traditional spelt	42	173	0.2	1.4	173	49.4	
PI 348478	2019-2020	traditional spelt	54	124	0.4	2.2	167	44.8	875
PI 348478	2020-2021	traditional spelt	60	107	0.6	2.6	188	50.5	
PI 348480	2019-2020	traditional spelt	75	118	0.6	3.1	258	53.1	-

Genotypes	Season	Type	P (mm)	L (mm)	P/L (ratio)	P/G (ratio)	W ($\times10^{-4}$J)	Ie (%)	Loaf Volume (cc)
PI 348480	2020-2021	traditional spelt	64	142	0.5	2.4	279	59.8	
PI 348483	2019-2020	traditional spelt	63	129	0.5	2.5	224	51.5	845
PI 348483	2020-2021	traditional spelt	67	119	0.6	2.8	230	52.4	
PI 348489	2019-2020	traditional spelt	41	111	0.4	1.7	116	43.3	805
PI 348489	2020-2021	traditional spelt	48	157	0.3	1.7	184	48.6	
PI 348493	2019-2020	traditional spelt	56	136	0.4	2.2	198	49.2	860
PI 348493	2020-2021	traditional spelt	69	164	0.4	2.4	288	52.3	
PI 348515	2019-2020	traditional spelt	54	136	0.4	2.1	157	40.1	-
PI 348515	2020-2021	traditional spelt	68	114	0.6	2.9	196	44.6	
PI 348544	2019-2020	traditional spelt	77	121	0.6	3.1	269	54.1	830
PI 348544	2020-2021	traditional spelt	72	120	0.6	3.0	257	55.2	
PI 348570	2019-2020	traditional spelt	69	97	0.7	3.2	176	42.9	725
PI 348570	2020-2021	traditional spelt	43	125	0.3	1.7	119	39.6	
PI 348572	2019-2020	traditional spelt	87	88	1.0	4.2	233	49.5	-
PI 348572	2020-2021	traditional spelt	63	117	0.5	2.6	206	50.6	
PI 348676	2019-2020	traditional spelt	64	109	0.6	2.8	169	40.8	760
PI 348676	2020-2021	traditional spelt	52	136	0.4	2.0	155	40.3	
PI 348696	2019-2020	traditional spelt	62	106	0.6	2.7	168	43.5	-
PI 348696	2020-2021	traditional spelt	65	117	0.6	2.7	202	47.9	
PI 348698	2019-2020	traditional spelt	60	95	0.6	2.8	153	43.0	-
PI 348698	2020-2021	traditional spelt	55	134	0.4	2.1	177	45.3	
PI 348701	2019-2020	traditional spelt	55	83	0.7	2.7	120	38.1	815
PI 348701	2020-2021	traditional spelt	51	119	0.4	2.1	148	42.7	

Genotypes	Season	Type	P (mm)	L (mm)	P/L (ratio)	P/G (ratio)	W (×10^{-4}J)	Ie (%)	Loaf Volume (cc)
PI 348712	2019-2020	traditional spelt	80	100	0.8	3.6	235	50.2	-
PI 348712	2020-2021	traditional spelt	53	134	0.4	2.1	188	49.5	
PI 348727	2019-2020	traditional spelt	35	103	0.3	1.5	83	36.2	-
PI 348727	2020-2021	traditional spelt	43	141	0.3	1.6	148	46.8	
PI 348741	2019-2020	traditional spelt	64	114	0.6	2.7	197	47.9	780
PI 348741	2020-2021	traditional spelt	61	129	0.5	2.4	206	50.1	
PI 348747	2019-2020	traditional spelt	48	166	0.3	1.7	164	42.8	863
PI 348747	2020-2021	traditional spelt	46	186	0.3	1.5	194	49.1	
PI 348767	2019-2020	traditional spelt	55	135	0.4	2.1	194	49.1	-
PI 348767	2020-2021	traditional spelt	55	158	0.4	2.0	210	49.2	
PI 348771	2019-2020	traditional spelt	47	141	0.3	1.8	129	36.6	740
PI 348771	2020-2021	traditional spelt	48	149	0.3	1.8	146	40.4	
PI 469022	2019-2020	traditional spelt	56	135	0.4	2.2	177	44.0	-
PI 469022	2020-2021	traditional spelt	51	137	0.4	2.0	156	42.2	
PI 469023	2019-2020	traditional spelt	67	133	0.5	2.6	223	47.8	760
PI 469023	2020-2021	traditional spelt	72	123	0.6	2.9	240	50	
PI 469024	2019-2020	traditional spelt	52	146	0.4	1.9	184	46.9	-
PI 469024	2020-2021	traditional spelt	40	172	0.2	1.4	153	45.5	
PI 469026	2019-2020	traditional spelt	58	133	0.4	2.3	202	49.3	775
PI 469026	2020-2021	traditional spelt	65	111	0.6	2.8	207	50.2	
PI 469028	2019-2020	traditional spelt	62	123	0.5	2.5	186	44.6	-
PI 469028	2020-2021	traditional spelt	66	108	0.6	2.9	200	49.5	
PI 469029	2019-2020	traditional spelt	42	106	0.4	1.8	93	32.6	-

Genotypes	Season	Type	P (mm)	L (mm)	P/L (ratio)	P/G (ratio)	W ($\times 10^{-4}$J)	Ie (%)	Loaf Volume (cc)
PI 469029	2020-2021	traditional spelt	39	109	0.4	1.7	94	35.9	
PI 469030	2019-2020	traditional spelt	56	140	0.4	2.1	173	41.8	-
PI 469030	2020-2021	traditional spelt	57	154	0.4	2.1	206	47.7	
PI 469031	2019-2020	traditional spelt	44	168	0.3	1.5	141	39.0	-
PI 469031	2020-2021	traditional spelt	40	151	0.3	1.5	128	41.5	
PI 469032	2019-2020	traditional spelt	39	135	0.3	1.5	104	36.4	710
PI 469032	2020-2021	traditional spelt	39	123	0.3	1.6	110	40.5	
PI 469034	2019-2020	traditional spelt	34	142	0.2	1.3	86	32.5	-
PI 469034	2020-2021	traditional spelt	39	107	0.4	1.7	98	38.5	
PI 469039	2019-2020	traditional spelt	50	129	0.4	2.0	149	42.4	850
PI 469039	2020-2021	traditional spelt	56	118	0.5	2.3	181	49.2	
PI 469040	2019-2020	traditional spelt	53	128	0.4	2.1	150	40.0	-
PI 469040	2020-2021	traditional spelt	64	109	0.6	2.8	182	44.6	
PI 469041	2019-2020	traditional spelt	70	110	0.6	3.0	242	56.2	870
PI 469041	2020-2021	traditional spelt	67	149	0.5	2.5	262	51.4	
PI 469042	2019-2020	traditional spelt	50	68	0.7	2.7	85	28.4	610
PI 469042	2020-2021	traditional spelt	41	122	0.3	1.7	117	41.2	
PI 469045	2019-2020	traditional spelt	59	119	0.5	2.4	166	41.8	-
PI 469045	2020-2021	traditional spelt	59	55	1.1	3.6	98	31.6	
PI 469046	2019-2020	traditional spelt	56	110	0.5	2.4	149	41.2	810
PI 469046	2020-2021	traditional spelt	56	123	0.5	2.3	162	42.4	
PI 469047	2019-2020	traditional spelt	41	141	0.3	1.6	109	35.2	710
PI 469047	2020-2021	traditional spelt	29	162	0.2	1.0	73	29.9	

Genotypes	Season	Type	P (mm)	L (mm)	P/L (ratio)	P/G (ratio)	W ($\times 10^{-4}$J)	Ie (%)	Loaf Volume (cc)
PI 469048	2019-2020	traditional spelt	51	121	0.4	2.1	138	39.4	790
PI 469048	2020-2021	traditional spelt	50	157	0.3	1.8	171	43.9	
PI 469049	2019-2020	traditional spelt	64	120	0.5	2.6	197	46.5	815
PI 469049	2020-2021	traditional spelt	76	111	0.7	3.2	241	50.4	
PI 469050	2019-2020	traditional spelt	63	119	0.5	2.6	202	48.7	870
PI 469050	2020-2021	traditional spelt	63	165	0.4	2.2	283	56.1	
PI 469051	2019-2020	traditional spelt	60	139	0.4	2.3	204	47.2	848
PI 469051	2020-2021	traditional spelt	66	150	0.4	2.4	264	53	
PI 469053	2019-2020	traditional spelt	49	132	0.4	1.9	162	46.5	795
PI 469053	2020-2021	traditional spelt	57	139	0.4	2.2	230	56	
PI 469054	2019-2020	traditional spelt	73	120	0.6	3.0	258	54.1	-
PI 469054	2020-2021	traditional spelt	76	122	0.6	3.1	280	55.3	
PI 469056	2019-2020	traditional spelt	72	79	0.9	3.6	154	38.2	775
PI 469056	2020-2021	traditional spelt	67	89	0.8	3.2	147	36.2	
PI 469057	2019-2020	traditional spelt	72	94	0.8	3.3	175	40.3	895
PI 469057	2020-2021	traditional spelt	94	81	1.2	4.7	224	44.3	
PI 469058	2019-2020	traditional spelt	58	149	0.4	2.1	209	48.7	885
PI 469058	2020-2021	traditional spelt	77	110	0.7	3.3	242	50.9	
PI 469059	2019-2020	traditional spelt	47	124	0.4	1.9	136	41.8	840
PI 469059	2020-2021	traditional spelt	62	107	0.6	2.7	206	55.4	
PI 469060	2019-2020	traditional spelt	43	141	0.3	1.6	127	39.9	758
PI 469060	2020-2021	traditional spelt	42	171	0.3	1.4	144	41.2	
Anna Maria	2019-2020	modern spelt	52	108	0.5	2.3	148	43.9	-

Genotypes	Season	Type	P (mm)	L (mm)	P/L (ratio)	P/G (ratio)	W (×10^{-4}J)	Ie (%)	Loaf Volume (cc)
Anna Maria	2020-2021	modern spelt	58	133	0.4	2.3	236	58.5	
Antequera	2019-2020	common wheat	106	120	0.9	4.3	421	61.9	815
Antequera	2020-2021	common wheat	127	103	1.2	5.6	437	59.5	
Conil	2019-2020	common wheat	145	82	1.8	7.2	448	63.9	-
Conil	2020-2021	common wheat	164	75	2.2	8.5	452	59.8	
Galera	2019-2020	common wheat	150	103	1.5	6.6	666	78.4	-
Galera	2020-2021	common wheat	145	108	1.3	6.3	653	77.6	
Motemayor	2019-2020	common wheat	118	94	1.3	5.5	402	62.8	785
Motemayor	2020-2021	common wheat	131	75	1.8	6.8	356	58.1	
Rota	2019-2020	common wheat	156	76	2.1	8.0	482	69.4	750
Rota	2020-2021	common wheat	197	55	3.6	11.9	458	65.5	
Setenil	2019-2020	common wheat	172	66	2.6	9.5	436	59.4	-
Setenil	2020-2021	common wheat	148	79	1.9	7.5	413	56.9	
Tejada	2019-2020	common wheat	104	82	1.3	5.1	253	45.0	765
Tejada	2020-2021	common wheat	119	71	1.7	6.3	251	40.4	

P, dough tenacity; L, dough extensibility; G: swelling index; W, dough strength; and Ie, elasticity index.

Table S4. Effects of genotype, season and genotype x season (GxS) on quality traits in spelt accessions. Sum of squares, % of the total sum of squares from ANOVA analysis and coefficient of variation (CV) are indicated.

Trait	Genotype Sum of squares (%)	Season Sum of squares (%)	GxS Sum of squares (%)	Error Sq. sum (%)	CV
	Grain/Flour components				
TW (Kg/hL)	591.7*** (49.0%)	144.6*** (12.0%)	185.9ns (15.4%)	284.9 (23.6%)	1.74
TKW (g)	6800.3*** (82.8%)	479.4*** (5.8%)	361.5ns (4.4%)	572.7 (7.0%)	3.62
GPC (%)	468.0*** (42.5%)	23.2** (2.1%)	158ns (14.3%)	450.7 (41%)	10.6
Hardness (%)	35380.4*** (82.1%)	3210.0*** (7.4%)	1799.2ns (4.2%)	2717.5 (6.3%)	26.1
Flour yield (%)	2531.4*** (66.5%)	408.2*** (10.7%)	866.9*** (22.8%)	0 (0%)	2.67
FPC (%)	251.6*** (73.7%)	4.9*** (1.4%)	84.95*** (24.9%)	0 (0%)	4.78
SDS-sed (ml)	2517.5*** (77.1%)	289.4*** (8.9%)	458.8*** (14.0%)	0 (0%)	8.28
PPO activity ($Ug^{-1}min^{-1}$)	1444.7*** (95.7%)	12.6*** (0.8%)	53.0*** (3.5%)	0 (0%)	4.91
	Alveogram parameters				
P (mm)	35976.1*** (83.3%)	672.4** (1.6%)	0ns (0%)	6552.6 (15.2%)	15.33
L (mm)	61881.1*** (72.3%)	1890.6* (2.2%)	0ns (0%)	21863.4 (25.5%)	13.43
P/L (ratio)	7.3*** (77.1%)	0.1ns (0.6%)	0ns (0%)	2.1 (22.3%)	31.72
P/G (ratio)	102.7*** (81.1%)	0.9ns (0.7%)	0ns (0%)	23.1 (18.2%)	21.89
W (x 10^{-4} J)	439088.0*** (84.9%)	31025.0*** (6.0%)	0ns (0%)	47154.0 (9.1%)	13.1
Ie (%)	5435.3*** (82.0%)	484.8*** (7.3%)	0ns (0%)	706.0 (10.7%)	6.48

TW, test weight; TKW, thousand kernel weight; GPC, grain protein content; FPC , flour protein contents; SDS-sed, Sodium dodecyl sulfate sedimentation test; PPO activity, polyphenol oxidase activity; P, dough tenacity; L, dough extensibility; G: swelling index; W, dough strength; and Ie, elasticity index.
***, **, *: significant at 99.9, 99 and 95%; ns: not significant.

Table S5. Correlation analysis between quality traits in spelt.

	TW	TKW	GPC	Hardness	Flour yield	FPC	SDS-sed	PPO activity	P	L	P/L	P/G	W
TKW	-0.261**												
GPC	-0.423***	0.305**											
Hardness	0.161	-0.130	0.047										
Flour yield	0.078	0.163	0.052	0.159									
FPC	-0.316**	0.220*	0.640***	0.067	0.217								
SDS-sed	-0.200	0.079	0.446***	-0.085	0.225*	0.800***							
PPO activity	0.011	-0.175	-0.019	-0.223*	-0.025	0.040	-0.012						
P	0.112	-0.108	-0.159	0.600***	-0.009	-0.093	-0.007	-0.154					
L	-0.109	-0.043	0.260*	-0.443***	0.127	0.374***	0.551***	0.196	-0.517***				
P/L	0.095	-0.022	-0.152	0.654***	-0.036	-0.185	-0.223*	-0.200	0.917***	-0.761***			
P/G	0.104	-0.059	-0.163	0.645***	-0.027	-0.156	-0.146	-0.185	0.970***	-0.683***	0.986***		
W	0.161	-0.258*	-0.179	0.366***	0.036	0.007	0.261*	-0.026	0.837***	-0.067	0.578***	0.694***	
Ie	0.229*	-0.401***	-0.254*	0.159	0.044	-0.044	0.266*	0.075	0.541***	0.136	0.262	0.380***	0.875***

TW, test weight; TKW, thousand kernel weight; GPC, grain protein content; FPC , flour protein contents; SDS-sed, Sodium dodecyl sulfate sedimentation test; PPO activity, polyphenol oxidase activity; P, dough tenacity; L, dough extensibility; G: swelling index; W, dough strength; and Ie, elasticity index.
***, **, *: significant at 99.9, 99 and 95%.
